# Eosinophils and pleural macrophages counter regulate IL-33-elicited airway inflammation via the 12/15-lipoxygenase pathway

**DOI:** 10.3389/fimmu.2025.1565670

**Published:** 2025-04-17

**Authors:** Emi Ito, Reika Hayashizaki, Takuro Hosaka, Tsuyoshi Yamane, Jun Miyata, Yosuke Isobe, Makoto Arita

**Affiliations:** ^1^ Division of Physiological Chemistry and Metabolism, Keio University Faculty of Pharmacy, Tokyo, Japan; ^2^ Laboratory for Metabolomics, RIKEN Center for Integrative Medical Sciences, Kanagawa, Japan; ^3^ Division of Pulmonary Medicine, Department of Medicine, Keio University School of Medicine, Tokyo, Japan; ^4^ Cellular and Molecular Epigenetics Laboratory, Graduate School of Medical Life Science, Yokohama City University, Kanagawa, Japan

**Keywords:** eosinophils, pleural macrophages, 12/15-lipoxygenase, IL-33, lipidomics

## Abstract

**Introduction:**

Fatty acid metabolism plays a crucial role in regulating airway inflammation through the synthesis of lipid mediators. We have previously demonstrated that a 12/15-lipoxygenase (12/15-LOX or Alox15)-derived mediator attenuates IL-33-induced eosinophilic airway inflammation in mice. However, the cellular sources of these mediators remain unclear.

**Methods:**

To identify the cellular sources, we used several cell type-specific conditional 12/15-LOX-deficient mice.

**Results:**

We found that eosinophils and pleural macrophages were the major 12/15-LOX-expressing cell types responsible for attenuating airway inflammation. Eosinophils were the major population of 12/15-LOX-expressing cells found in inflamed lung tissue. In addition, pleural macrophages were the major population of 12/15-LOX-expressing cells in the thoracic cavity and were found to translocate into inflamed lung tissue in response to airway inflammation.

**Discussion:**

This study suggests that eosinophils and pleural macrophages cooperatively regulate eosinophilic airway inflammation via 12/15-LOX expression. Targeting 12/15-LOX metabolism in these cells may offer new therapeutic strategies for severe asthma.

## 1 Introduction

Asthma is a chronic airway disease that affects approximately 300 million people worldwide (1). In asthma, eosinophilic airway inflammation is induced by type 2 cytokines released from Th2 cells and type 2 innate lymphoid cells (ILC2) when exposed to allergens, air pollutants, and microorganisms (2). IL-33 is a cytokine released by airway epithelial cells in response to airway damage, activating ILC2 and producing abundant type 2 cytokines that amplify eosinophilic inflammation (3, 4). IL-33 is also known as a potent activator of eosinophils, primarily inducing the phosphorylation of NF-kB signaling-related proteins (5, 6). IL-33 and its receptor IL1RL1 have been identified as causative molecules associated with asthma and eosinophilia in the Genome-wide Associated Studies (GWAS) database, suggesting a close relationship between these pathophysiologies (7–9).

Polyunsaturated fatty acids such as arachidonic acid (AA), docosahexaenoic acid (DHA) and eicosapentaenoic acid (EPA) are released from membrane phospholipids upon inflammatory stimuli and are then converted into bioactive lipid mediators by fatty acid oxygenases. 5-lipoxygenase (LOX) and cyclooxygenase (COX) mediate the biosynthesis of leukotrienes (LTs) and prostaglandins (PGs), respectively (10, 11). 15-LOX promotes the conversion of AA into lipoxins (LXs) and also promotes conversion of n-3 fatty acids (such as DHA and EPA) into resolvins (Rv), protectins, and maresins (MaR), which are specialized pro-resolving mediators (SPMs) that play a role in promoting anti-inflammatory phenotypes (12, 13).

In humans, 15-LOX (or ALOX15) is highly expressed in eosinophils, macrophages, and epithelial cells (14–16). We have previously demonstrated that 15-LOX activity was impaired in peripheral blood eosinophils obtained from patients with severe asthma and in nasal polyp-derived eosinophils obtained from patients with eosinophilic chronic rhinosinusitis (14, 17). In addition, 15-LOX-derived mediators such as LXA_4_ and protectin D1 suppressed cellular activation of human eosinophils and ILC2, suggesting that impaired 15-LOX activity may attenuate eosinophilic inflammation (14, 18, 19). Indeed, genetic deletion of 12/15-LOX, a mouse ortholog of 15-LOX, resulted in a severe phenotype of IL-33-induced eosinophilic airway inflammation in mice (20). Although 12/15-LOX plays a suppressive role in eosinophilic airway inflammation, the specific cell types that contribute to this regulatory function remain unknown.

In the present study, we examined the cell types responsible for local production of 12/15-LOX-derived mediators by using cell type-specific 12/15-LOX-deficient mice and found that eosinophils and pleural macrophages were the major cell types responsible for regulating airway inflammation via local production of 12/15-LOX-derived mediators *in vivo*.

## 2 Materials and methods

### 2.1 Animal preparations

Male mice (7- to 11-week-old) with a C57BL6/J background were housed under specific pathogen-free conditions in accordance with the guidelines of the Institutional Animal Care Committee of the RIKEN Yokohama Institute. Eo-Cre (21), LysM-Cre (22), Mcpt8-Cre (23), CD11c-Cre (24), Sftpc-CreER^T2^ (25), Scgb1a1-CreER^TM^ (26) mice were crossed with Alox15 floxed mice. Eo-Cre mice were kindly provided by Dr. J. Lee (Mayo Clinic, Arizona, USA). Alox15-Cre; Ai14 mice were generated by crossing Ai14 mice (ROSA26-loxP-stop-loxP-tdTomato reporter mice, obtained from the Jackson Laboratory) with Alox15-Cre mice. In the Alox15-Cre mice, Cre recombinase was inserted into the Alox15 gene locus to replace the endogenous translational start codon, followed by a transcriptional stop polyA sequence. The Alox15-Cre genome sequence was created by Dr. M. Nakayama (Kazusa DNA Institute Advanced Research and Development Department from the Omics Medical Science Laboratory). To induce Cre-mediated recombination in CreER^T2^ and Scgb1a1-CreER^TM^ mice, tamoxifen (Sigma-Aldrich, Taufkirchen, Germany) was dissolved in 150 µl of EtOH and corn oil at a concentration of 20 mg/ml and was administered by intraperitoneal administration for three consecutive days (9 mg in total).

### 2.2 Establishment of IL-33-induced airway inflammation

Recombinant mouse IL-33 protein (R&D Systems, Minneapolis, MN, USA; 500 ng per mouse) was reconstituted in 40 µL PBS. Next, this IL-33 was administered intranasally under intraperitoneal administration of either ketamine (100 mg/kg) and xylazine (10 mg/kg), or isoflurane as anesthetic agents for three consecutive days, and the mice were examined four days after the last challenge. Four days prior to the IL-33 administration, the mice were injected intrathoracically with 100 µL of 10 µM PKH26PCL fluorescent dye (Sigma Aldrich).

### 2.3 Collection of cells from bronchoalveolar lavage fluid and pleural lavage fluid

The trachea was cannulated and the lungs were lavaged by washing twice with 0.7 mL of ice-cold PBS containing 0.6 mM EDTA. After making a small incision through the diaphragm, 2.0 mL of ice-cold PBS containing 0.6 mM EDTA was injected into the pleural space. Pleural lavage fluid (PLF) was retrieved with a 2.5-mL syringe and maintained on ice. The total number of cells in Bronchoalveolar Lavage (BAL) or PLF was counted using a hemocytometer. Cells were stained with antibodies to C-C chemokine receptor type 3 (CCR3) FITC (83103; RD Systems, Minneapolis, MN, USA), PI (BioLegend), CD11b PE/Cy7 (M1/70; BD PharMingen), F4/80 APC (BM8; BioLegend), Siglec-F BV421 (E50-2440; BD PharMingen, San Jose, CA, USA), and CD45 BV510 (30-F11; BioLegend), and flow cytometric analysis was performed to estimate differential cell counts.

### 2.4 Adoptive transfer of pleural macrophages

Alox15-Cre; Ai14 mice were used for donor cells. tdTomato^+^ cells were isolated from PLF using FACS Aria II cell sorter (BD Biosciences). 2.9×10^5^ cells were resuspended in 100 µL of PBS and were injected into the pleural cavity of recipient C57BL6/J mice.

### 2.5 Analysis of cells from lung tissue by flow cytometry

Lungs were cut and incubated in RPMI 1640 (Gibco) containing 1 mg/mL each of collagenase II and DNase I at 37°C. Single-cell suspensions were blocked with an anti-mouse cluster of differentiation (CD) 16/32 blocking antibody (93; BioLegend, San Diego, CA, USA) and stained with CD24 PE (M1/69; BioLegend), CD64 PE/Cy7 (X54-5/7.1; BioLegend), CD11b APC (M1/70; BioLegend), Ghost Dye™ Red 780 (TONBO Biosciences) MHC2 BV421 (M5/114. 15.2; BioLegend), CD45 BV510 (30-F11; BioLegend). For 12/15-LOX staining, the cells were fixed and permeabilized using eBioscience™ Intracellular Fixation & Permeabilization Buffer Set (88-8824-00; Thermo Fisher Scientific, Waltham, MA, USA) and stained with rabbit anti-mouse 12/15-LOX antibody as primary antibody and then Alexa Fluor 488-conjugated (Thermo Fisher Scientific) donkey anti-rabbit IgG as secondary antibody. Flow cytometry was performed using FACS Aria II cell sorter (BD Biosciences), and FlowJo v. 9.9.6. software was used for analysis.

### 2.6 Lung histology

Lung slides were stained with Hematoxylin and Eosin (HE) or Periodic Acid Schiff (PAS) stain. As described previously (27), the quantity of perivascular or peribronchial inflammation was assessed, and a grade of 0 was assigned when no inflammation was detectable; grade 1 for occasional cuffing with inflammatory cells; and grades 2, 3, and 4 when most bronchi or vessels were surrounded by a thin layer (1–3 cells), moderate layer (3–5 cells), and thick layer (> 5 cells) of inflammatory cells, respectively. Total inflammation score was calculated by adding the peribronchial and perivascular inflammation scores.

### 2.7 Targeted liquid chromatography mass-spectrometry-based lipidomics

Lipid metabolites were extracted by solid-phase extraction using MonoSpin C18-AX cartridges (GL Science, Shinjuku, Tokyo, Japan) in the presence of the following deuterated internal standards: 1 ng each of arachidonic acid (AA)-d8, 15-hydroxyeicosatetraenoic acid (HETE)-d8, leukotriene B_4_ (LTB_4_)-d4, LTD_4_-d5, prostaglandin E_2_ (PGE_2_)-d4, PGB_2_-d4, and 8-iso-PGF2a-d4. For LC-MS/MS analysis, a triple-quadrupole linear ion-trap mass spectrometer (5500QTRAP; Sciex, Framingham, MA, USA or 4500QTRAP; Sciex, Framingham, MA, USA) equipped with an ACQUITY UPLC BEH C18 column (1.0 × 150 mm, 1.7-μm particle size; Waters, Milford, MA, USA) was used. Samples were eluted with a mobile phase composed of water/acetate (100:0.1, v/v) and acetonitrile/methanol (4:1, v/v) at a ratio of 73:27 for 5 min, ramped to 30:70 after 15 minutes, to 20:80 after 25 minutes and held for 10 minutes, to 5:95 after 35 min and held for 9 minutes, and to 0:100 after 39 min and held for 1 min. The flow rates were 50 μL/min (0-30 min), 80 μL/min (30-33 min), and 100 μL/min (33-40 min). MS/MS analyses were conducted in the negative ion mode, and lipid metabolites were identified and quantified by multiple reaction monitoring (MRM). Calibration curves between 1 and 1000 pg and LC retention times for each compound were established using synthetic standards.

### 2.8 Immunofluorescence

Lung tissues were embedded in OCT compound and frozen in liquid nitrogen. The embedded frozen sections were blocked using 1% bovine serum albumin (BSA) in PBS. The sections were then incubated with 0.5% rabbit anti-mouse 12/15-LOX antibody, 1% rat anti-mouse Siglec-F antibody (E50-2440; BD PharMingen), or 1% rabbit anti-mouse GATA6 antibody (D61E4; Cell Signaling Technology) overnight at 4°C. Next, the sections were incubated with secondary antibodies, such as 0.5% donkey anti-rabbit IgG antibody (Alexa Fluor 488), 0.5% donkey anti-rat IgG antibody (Alexa Fluor 488), 0.5% donkey anti-rat IgG antibody (Alexa Fluor 594), 0.5% F4/80 FITC antibody, and 0.25% DAPI at room temperature for 1 h. Images were captured using a fluorescence microscope (BZ-X700; Keyence, Tokyo, Japan).

### 2.9 Statistical analysis

Data are presented as mean ± standard error of the mean (SEM). Comparisons between animal groups were performed using one-way ANOVA with Dunnett’s *post hoc* test. Data were analyzed using GraphPad Prism version 9.1c (GraphPad Software, San Diego, CA, USA). P < 0.05 was considered statistically significant.

## 3 Results

### 3.1 Genetic deletion of 12/15-LOX in eosinophils and macrophages augmented IL-33-induced airway eosinophilic inflammation

**Figure 1:**
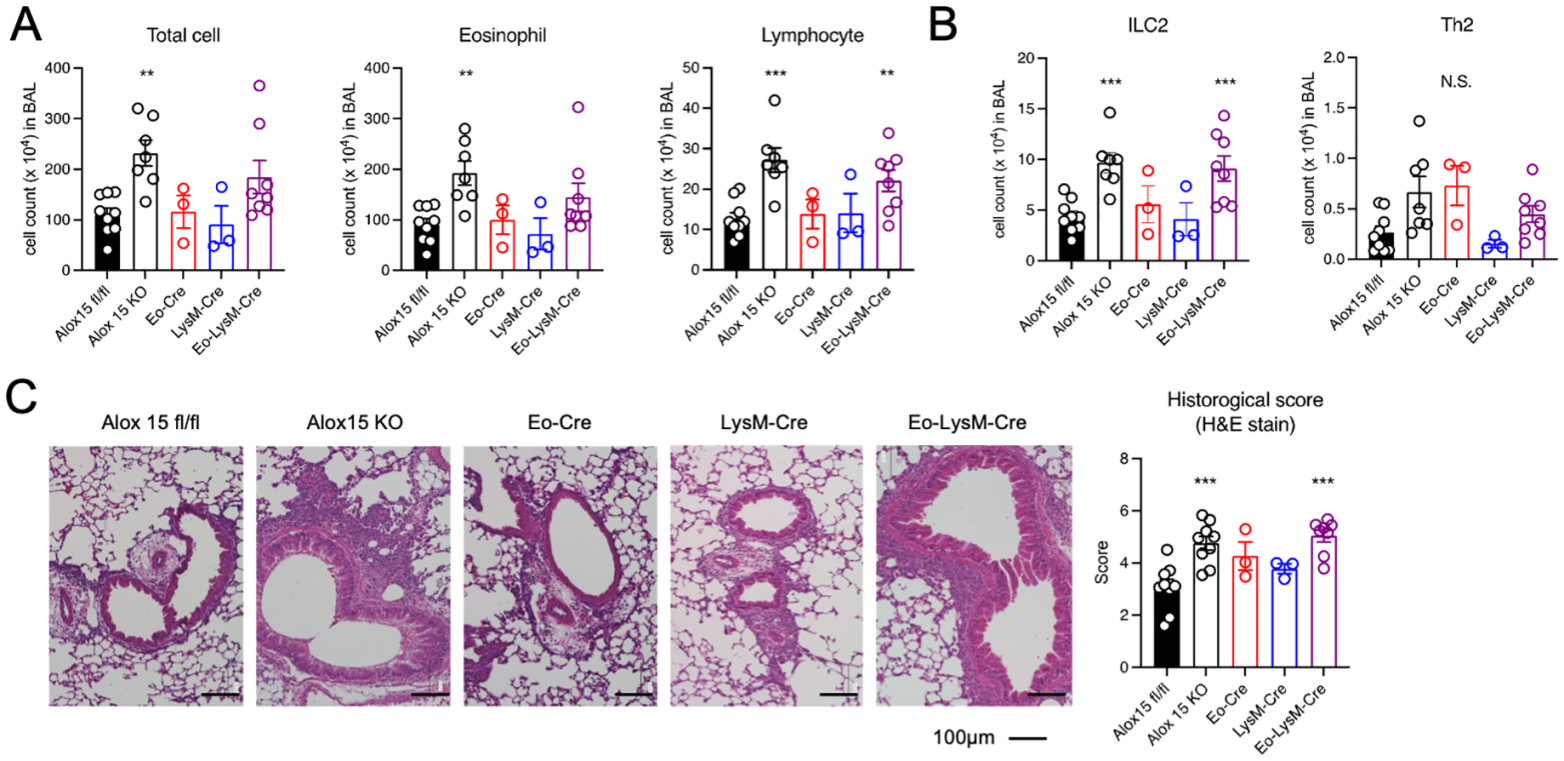
Genetic deletion of 12/15-LOX in eosinophils and macrophages augmented IL-33-induced airway eosinophilic inflammation. Airway inflammation was induced by administration of IL-33 in wild-type and 12/15-LOX-deficient mice (Alox15 KO) and cell type-specific conditional 12/15-LOX-deficient mice (Eo-Cre Alox15^fl/fl^: Eo-Cre, LysM-Cre Alox15^fl/fl^: LysM-Cre, Eo-Cre/LysM-Cre Alox15^fl/fl^: Eo-LysM-Cre). Analysis was carried out four days after the final administration of IL-33. **(A)** Number of total cells, eosinophils, and lymphocytes in bronchoalveolar lavage (BAL) fluid. **(B)** Number of ILC2 and Th2 cells in BAL obtained by flow cytometric analysis. **(C)** Hematoxylin and Eosin staining of lung tissue section. Data are pooled from three independent experiments. Mean ± SEM, n=3-9 for each group. **P<0.01 and ***P<0.001 compared with Alox15^fl/fl^ mice. N.S., not significant.

To examine the effects of different cell types expressing 12/15-LOX on eosinophilic airway inflammation, we generated cell type-specific conditional knockout mice. 12/15-LOX was deleted in eosinophils (Eo-Cre × Alox15^fl/fl^ mice), macrophages (LysM-Cre × Alox15^fl/fl^ mice), and in both eosinophils and macrophages (Eo-Cre/LysM-Cre × Alox15^fl/fl^ mice). Airway eosinophilic inflammation was induced by repeated intra nasal administration of IL-33. We first confirmed that no airway inflammation was observed in Alox15^fl/fl^ and systemic 12/15-LOX deficient mice following PBS administration (**Supplementary Figure 1**). While Eo-Cre × Alox15^fl/fl^ and LysM-Cre × Alox15^fl/fl^ mice showed an inflammatory response induced by IL-33 similar to that of the controls, Eo-Cre/LysM-Cre × Alox15^fl/fl^ mice exhibited exacerbated airway inflammation comparable to that of systemic 12/15-LOX-deficient mice (**Figure 1A**). The number of ILC2 in the BAL increased in Eo-Cre/LysM-Cre × Alox15^fl/fl^ and systemic 12/15-LOX deficient mice but not in Eo-Cre × Alox15^fl/fl^ or LysM-Cre × Alox15^fl/fl^ mice (**Figure 1B**). Similarly, Eo-Cre/LysM-Cre × Alox15^fl/fl^ mice exhibited an increased lung tissue inflammation score to the same extent as that of systemic 12/15-LOX deficient mice (**Figure 1C**).

We further established Cd11c-Cre × Alox15^fl/fl^, Mcpt8-Cre × Alox15^fl/fl^, Sftpc-Cre × Alox15^fl/fl^, and Scgb1a1-Cre × Alox15^fl/fl^ mice for conditional deletion of 12/15-LOX in dendritic cells, basophils, alveolar epithelial cells, and airway epithelial cells, respectively. These mice did not show any changes in airway inflammation (**Supplementary Figure 2**). These results demonstrate that eosinophils and certain macrophages are responsible for the enhanced airway inflammation observed in systemic 12/15-LOX deficient mice.

### 3.2 Synthesis of 12/15-LOX-derived metabolites in lung was impaired by genetic deletion of 12/15-LOX in eosinophils and macrophages

**Figure 2:**
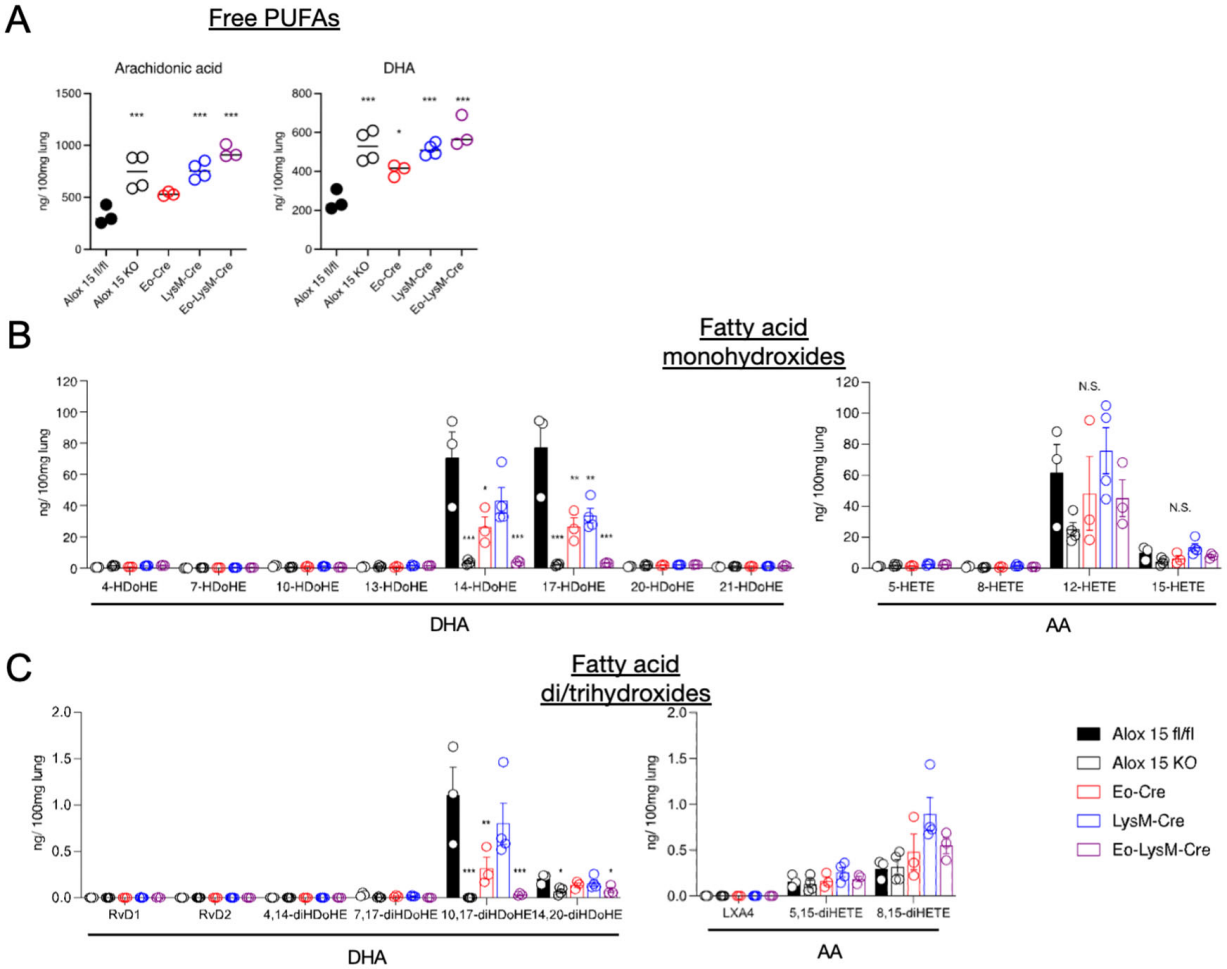
Genetic deletion of 12/15-LOX in eosinophils and macrophages resulted in impaired synthesis of 12/15-LOX-derived metabolites in the lungs. Airway inflammation was induced by administration of IL-33 in wild-type and 12/15-LOX-deficient mice (Alox15 KO) and cell type-specific conditional 12/15-LOX-deficient mice (Eo-Cre Alox15^fl/fl^: Eo-Cre, LysM-Cre Alox15^fl/fl^: LysM-Cre, Eo-Cre/LysM-Cre Alox15^fl/fl^: Eo-LysM-Cre). Analysis was carried out four days after the final administration of IL-33. Comparative analysis of amounts of **(A)** free polyunsaturated fatty acids [arachidonic acid (AA) and docosahexaenoic acid (DHA)] and **(B, C)** their metabolites, including bioactive lipid mediators such as lipoxin (LX) and resolvin (Rv), in wild-type or each of the 12/15-LOX-deficient mice. Mean ± SEM, n = 3-5 for each group. *P<0.05, **P<0.01, and ***P<0.001 compared with Alox15^fl/fl^ mice.

LC-MS/MS-based mediator lipidomics of inflamed lung tissue was performed. While the amounts of free polyunsaturated fatty acids (PUFAs) in the lung tissue were comparable in all groups, the amounts of 12/15-LOX metabolites, 14-Hydroxydocosahexaenoic acid (HDoHE), 17-HDoHE, 10,17-diHDoHE, and 14,20-HDoHE were partially reduced in Eo-Cre × Alox15^fl/fl^ or LysM-Cre × Alox15^fl/fl^ mice compared to those in the wild-type mice (**Figures 2A–C**, **Supplementary Table 1**). Notably, these metabolites further decreased in Eo-Cre/LysM-Cre × Alox15^fl/fl^ mice to the same extent as in systemic 12/15-LOX deficient mice (**Figures 2B, C**). No differences were observed in the levels of AA-derived dihydroxy and trihydroxy metabolites (**Figure 2C**). These results suggest that eosinophils and macrophages jointly contribute to the local production of 12/15-LOX metabolites in the lungs during IL-33-induced eosinophilic inflammation.

### 3.3 12/15-LOX-expressing cells in lung during IL-33-induced airway inflammation

**Figure 3:**
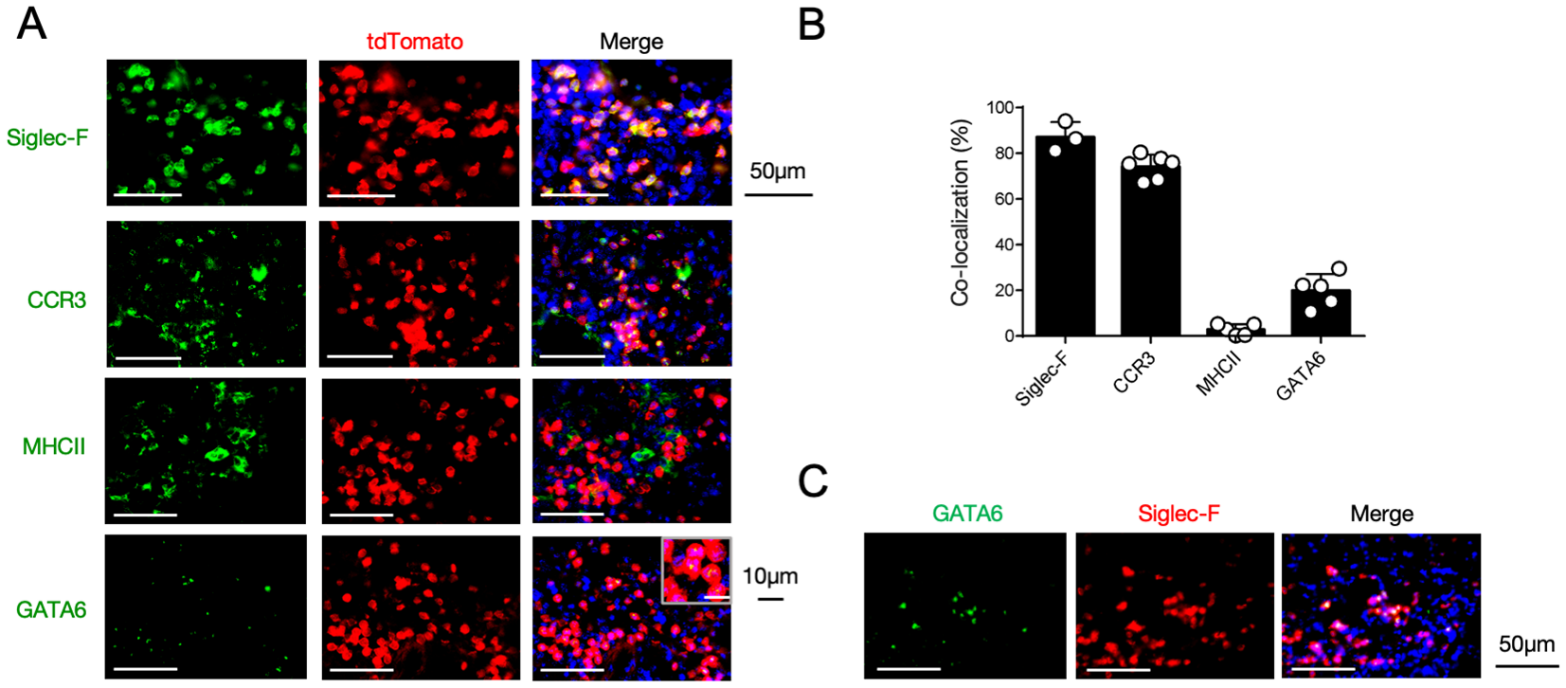
12/15-LOX-expressing cells in the lungs during IL-33-induced airway inflammation. **(A)** Immunofluorescence analysis of lung tissue in Alox15-Cre; Ai14 mice, four days after the final administration of IL-33. Fluorescence images of GATA6 (green), tdTomato (red), and DAPI (blue) are shown. **(B)** Percentage of each cell marker-positive and tdTomato^+^ cells among the total tdTomato^+^ cells obtained from immunofluorescence analysis of lung tissue (n = 3-6/group). **(C)** Immunofluorescence analysis of lung tissue in wild-type mice four days after the final administration of IL-33. Fluorescence images of GATA6 (green), Siglec-F (red), and DAPI (blue) are shown.

We next sought to identify 12/15-LOX-expressing cells in the inflamed lung tissue using 12/15-LOX reporter mice, in which tdTomato is expressed following Cre-mediated recombination under the control of the 12/15-LOX promoter. Immunohistochemical analysis revealed that fluorescence of the 12/15-LOX reporter co-localized with that of Siglec-F, which is typically expressed in eosinophils and alveolar macrophages in inflamed lung tissues (**Figures 3A, B**). While ~75% of 12/15-LOX-positive cells were also positive for the eosinophil marker CCR3, they rarely (< 5%) co-localized with MHC class II, which is highly expressed in alveolar macrophages (**Figures 3A, B**). Instead, ~20% of the 12/15-LOX signals overlapped with those of Gata6, a transcription factor that is highly expressed in cavity macrophages, including pleural macrophages (28) (**Figures 3A, B**). Gata6-positive cells also merged with a portion of the Siglec-F signals in the inflamed lung tissue (**Figure 3C**).

### 3.4 Pleural macrophages are 12/15-LOX-expressing LysM^+^ cells in the respiratory system

**Figure 4:**
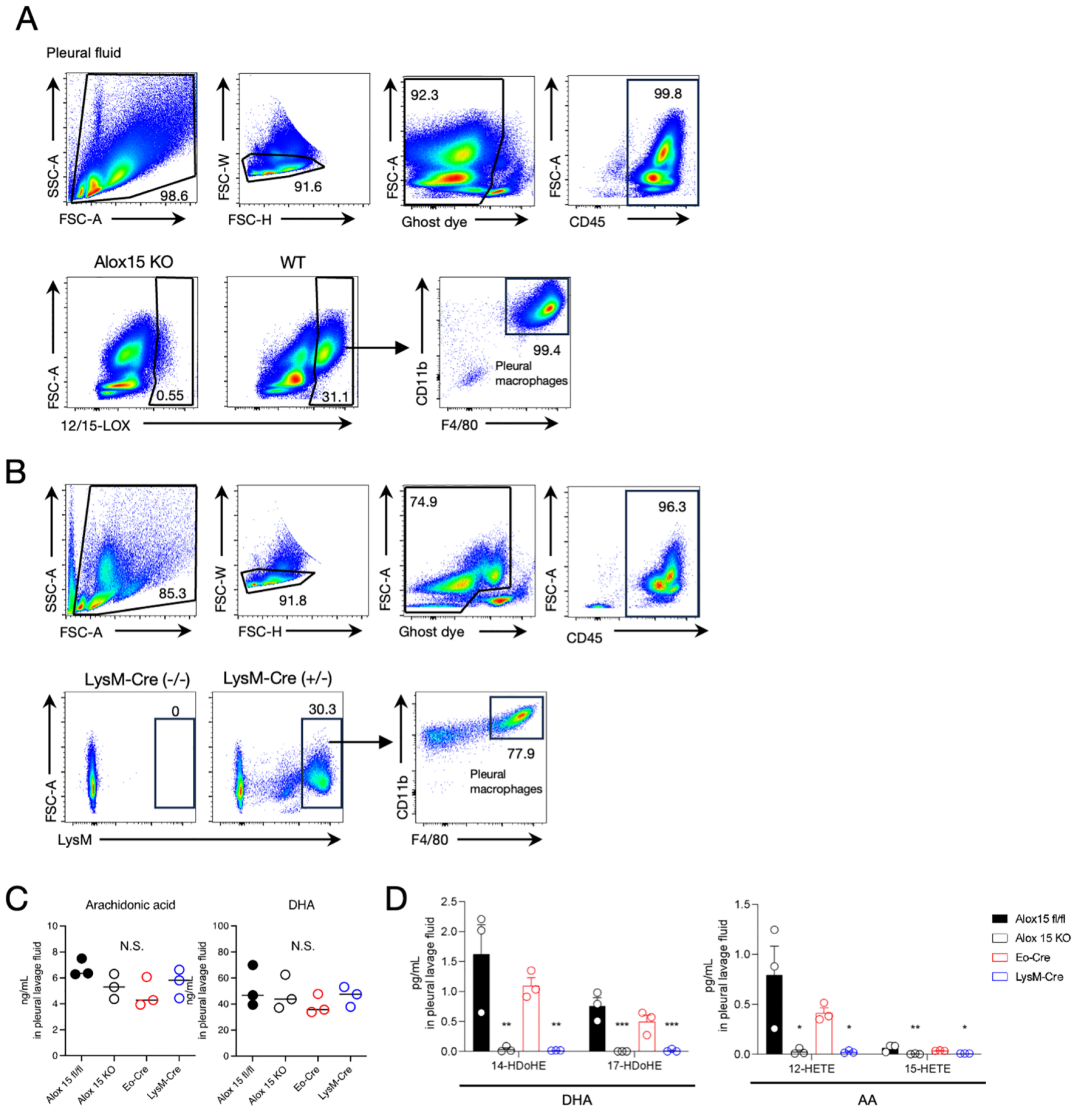
Pleural macrophages are 12/15-LOX-expressing LysM^+^ cells. **(A)** Flow cytometric analysis of 12/15-LOX-expressing cells in pleural lavage fluid at a steady state. **(B)** Flow cytometric analysis of LysM^+^ cells in pleural lavage fluid at a steady state in LysM-Cre; Ai14 mice. **(C, D)** The amounts of free polyunsaturated fatty acids [arachidonic acid (AA) and docosahexaenoic acid (DHA)] **(C)** or 12/15-LOX-derived monohydroxy metabolites **(D)** in the pleural lavage fluid four days after the final administration of IL-33. Mean ± SEM, n=3 for each group. *P<0.05, **P<0.01, and ***P<0.001 compared with Alox15^fl/fl^ mice. N.S., not significant.

The results mentioned above led us to hypothesize that macrophages in the thoracic cavity might contribute to the local production of 12/15-LOX metabolites in cooperation with eosinophils during airway inflammation. Flow cytometric analysis revealed that pleural macrophages exclusively expressed 12/15-LOX among the CD45^+^ cells in the thoracic cavity (**Figure 4A**). Using 12/15-LOX reporter mice, we also confirmed that more than 90% of tdTomato^+^ cells were pleural macrophages in the thoracic cavity (**Supplementary Figure 3A**). The expression level of 12/15-LOX in pleural macrophages was comparable to that in peritoneal macrophages, another subset of cavity macrophages known for their high 12/15-LOX expression (29, 30) (**Supplementary Figure 3B**). Also, 12/15-LOX expression in eosinophils was nearly half that of pleural macrophages (**Supplementary Figures 4A, B**). Flow cytometry analysis of cells of LysM reporter (LysM-Cre; Ai14) mice revealed that the majority of LysM^+^ cells in the thoracic cavity were pleural macrophages (**Figure 4B**). In addition, lipidomic analysis showed that 12/15-LOX metabolites (14-HDoHE, 17-HDoHE, 12-HETE, and 15-HETE) were abundantly present in the thoracic cavity, and the amounts of these metabolites were dramatically reduced in LysM-Cre × Alox15^fl/fl^ mice to the same extent as those in systemic 12/15-LOX deficient mice; however, no changes were observed in Eo-Cre × Alox15^fl/fl^ mice compared to the controls (**Figures 4C, D**, **Supplementary Table 2**).

### 3.5 IL-33 administration promotes translocation of pleural macrophages to the lungs

**Figure 5:**
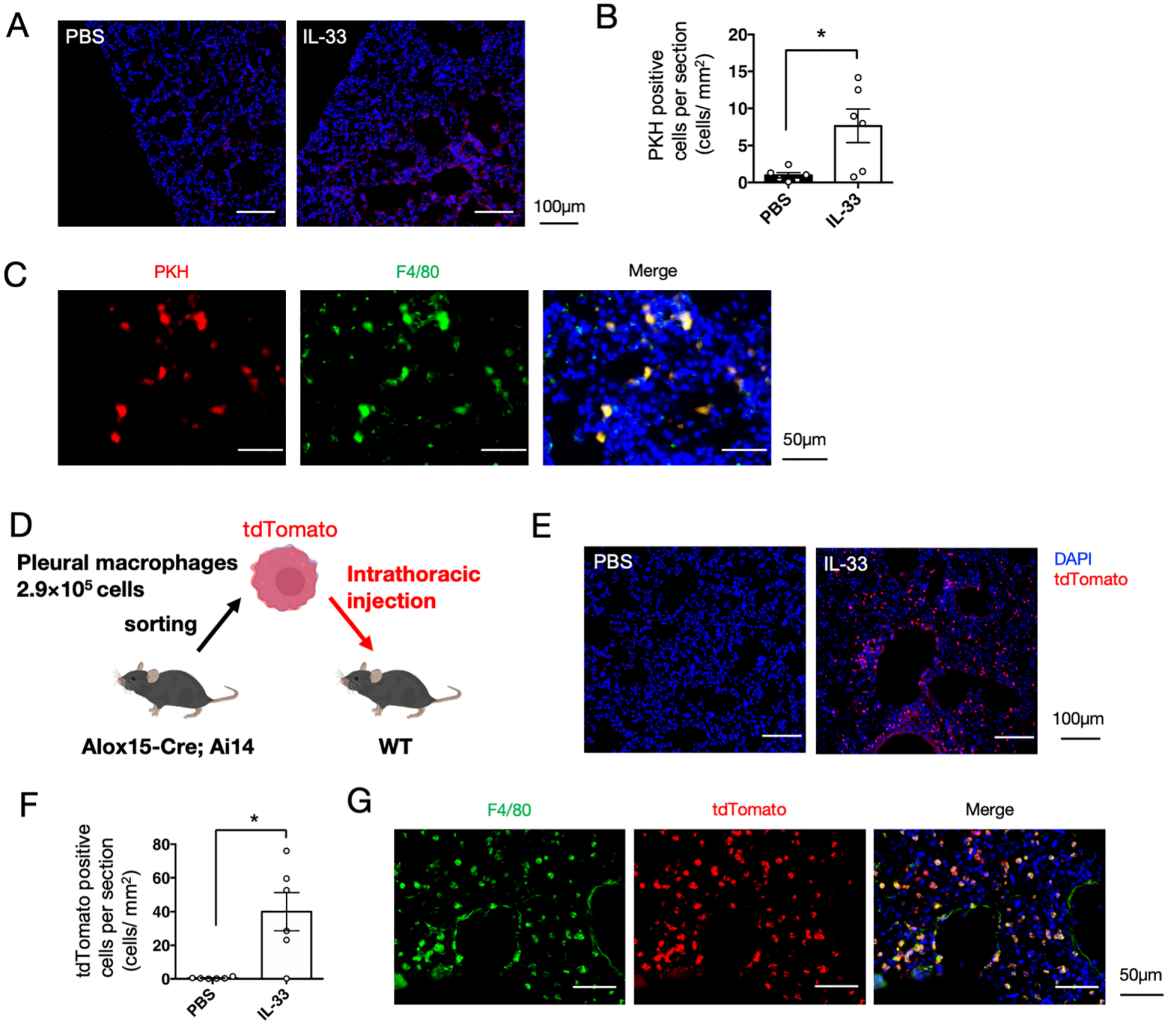
Pleural macrophages are translocated to lung tissue upon IL-33 administration. **(A)** Pleural macrophages were labeled with PKH26 dye before induction of airway inflammation. Representative fluorescent image of the lung tissue section four days after administration of PBS or IL-33. **(B)** Quantification of the number of PKH26^+^ cells per section (mm^2^) (n = 6/group). **(C)** Lung tissue section of PKH26-injected mice co-stained with a macrophage marker F4/80 is shown. **(D)** Schematic representation of adoptive cell transfer. Pleural macrophages obtained from lung tissues of 12/15-LOX reporter mice were injected into the thoracic cavity of wild-type mice, followed by induction of airway inflammation. **(E)** Representative fluorescent image of the lung tissue section of wild-type mice transferred with tdTomato^+^ pleural macrophages, four days after the final administration of IL-33 or PBS. **(F)** Quantification of the number of tdTomato^+^ cells per section (mm^2^) (n = 6/group). Mean ± SEM, *P<0.05. **(G)** Lung tissue section of a mouse transferred with tdTomato+ pleural macrophages, followed by IL-33 administration, was co-stained with F4/80.

Recent studies have demonstrated that cavity-resident macrophages, including pleural macrophages, migrate into tissues in response to viral infections and tissue damage (31, 32). Therefore, we next investigated whether pleural macrophages migrate into the lung tissue using a phagocyte-labeling dye PKH26 (31). Pleural macrophages were labeled *in vivo* by intrathoracic injection of PKH26 dye four days prior to IL-33 administration. PKH26-positive cells were observed in the lungs, and upon IL-33 administration, their number increased significantly than in the control group (**Figures 5A, B**). The PKH26-positive cells were observed to be particularly abundant around the bronchi (**Figure 5A**). In addition, PKH26 signals colocalized with the macrophage marker F4/80 in lung tissue (**Figure 5C**). To further confirm the recruitment of pleural macrophages, we isolated tdTomato^+^ cells from the thoracic cavity of 12/15-LOX reporter mice and injected them into the thoracic cavity of recipient wild-type mice, followed by administration of IL-33 (**Figure 5D**). tdTomato^+^ cells were detected in the lungs using histological imaging, and their number was significantly increased upon IL-33 administration (**Figures 5E, F**). Consistent with the cell-tracing experiment using the PKH26 dye, tdTomato^+^ cells in lung tissue expressed the macrophage marker F4/80 (**Figure 5G**). We also confirmed the expression of Siglec-F and Gata6 in tdTomato^+^ cells within the inflamed lung tissue (**Supplementary Figure 5**). Collectively, these results suggest that pleural macrophages translocate to the lungs upon induction of airway inflammation.

## 4 Discussion

In this study, we identified eosinophils and LysM^+^ macrophages as cell types expressing 12/15-LOX responsible for controlling eosinophilic airway inflammation using a number of cell type-specific conditional 12/15-LOX deficient mice. The expression of 12/15-LOX co-localized with that of an eosinophil marker CCR3 and cavity macrophage marker Gata6 in inflamed lungs. Pleural macrophages are cavity macrophages present in the respiratory system that are LysM^+^ and exclusively express 12/15-LOX in the thoracic cavity. In addition, cell-tracing experiments revealed that pleural macrophages translocate to the lung tissue upon induction of airway inflammation.

12/15-LOX is a fatty acid oxygenase that is involved in the production of anti-inflammatory lipid metabolites, collectively called specialized pro-resolving mediators (SPMs) (12). Murine models of asthma have demonstrated suppressive effects of 12/15-LOX-derived mediators on eosinophilic inflammation (20, 33–37). In humans, the enzymatic activity of 15-LOX, an ortholog of the mouse 12/15-LOX, was reduced in severe asthma, implying that 15-LOX-derived SPMs contribute towards the pathogenesis of chronic eosinophilic inflammation in severe asthma (14). In addition, these mediators exert pro-resolving effects on established inflammation when they are administered after exposure to antigens (33, 35–37). This study demonstrated that eosinophils and pleural macrophages are responsible for the local production of 12/15-LOX-derived metabolites in IL-33-induced airway inflammation. It has been previously shown that diverse sets of lipid mediators can be generated through the 12/15-LOX pathway. For example, AA is oxidized by 12/15-LOX to form 15-Hydroperoxyeicosatetraenoic acid (HpETE), which is further metabolized to LXA_4_ through a 5-LOX-catalyzing reaction. 15-HpETE can also be metabolized to eoxins through the biosynthetic pathway of cysteinyl leukotriene (cysLTs) (38). 12/15-LOX metabolizes not only AA but also EPA and DHA to form a diverse array of SPMs (39–41). A complex lipid mediator environment is created at inflammatory sites, which is shaped by the lipid metabolic features of each cell type present at these sites. Studies on deeper profiles of these lipid mediators related to the course of inflammation will uncover the specific roles of eosinophils and pleural macrophages in the production of 12/15-LOX-derived mediators, as well as the pathophysiology of eosinophilic airway inflammation regulated by them.

Excessive tissue accumulation of eosinophils exacerbates inflammatory reactions and tissue damage in allergic disorders, including asthma (42). On the other hand, eosinophils are known to be involved in the maintenance of tissue homeostasis (43–46). In the lungs, tissue-resident eosinophils have been shown to play a role in suppressing allergen-mediated eosinophil and pathogen-induced neutrophilic inflammation (44, 45). However, the molecular mechanisms by which eosinophils suppress allergic airway inflammation have not yet been fully elucidated. In the current study, we showed that eosinophils suppressed airway inflammation via 12/15-LOX, a fatty acid oxygenase that is highly expressed in eosinophils. We previously demonstrated that 12/15-LOX-expressing eosinophils enhanced clearance of apoptotic neutrophils via biosynthesis of PD1 and LXA_4_, thereby resolving neutrophilic inflammation (40, 47, 48). Additionally, a report suggested that eosinophils promote corneal epithelial wound healing via 12/15-LOX activity to produce 17-HDoHE in the cornea (49). Taken together, these data indicate that eosinophils may suppress airway inflammation possibly through the formation and action of 12/15-LOX-derived pro-resolving mediators.

In the present study, we found that pleural macrophages were the major cell type that expressed 12/15-LOX in the thoracic cavity. Pleural macrophages are resident cells in the thoracic cavity that are responsible for phagocytosis of apoptotic cells to maintain homeostasis in the respiratory system (50, 51). Expression patterns of surface antigens and profiles of cytokine production in pleural macrophages stimulated with LPS are similar to those in peritoneal macrophages (52, 53). Peritoneal macrophages phagocytose and clear apoptotic cells in a 12/15-LOX-dependent manner, and are responsible for maintaining homeostasis in the peritoneal environment (30). The expression levels of 12/15-LOX in pleural and peritoneal macrophages were almost similar (**Supplementary Figure 3B**). Thus, 12/15-LOX may be a key lipid-metabolizing enzyme that confers anti-inflammatory and tissue-protective functions on pleural macrophages to maintain tissue homeostasis. Emerging evidence suggests that cavity macrophages translocate to the surrounding tissues. For example, pleural macrophages migrate to the lungs upon influenza virus infection (31). Peritoneal macrophages are recruited to injured liver in response to damage-associated molecular pattern signals (DAMPs) and promote tissue repair (32). This study highlights the multifunctional roles of cavity macrophages in maintaining homeostasis of surrounding tissues by producing 12/15-LOX-derived lipid mediators. However, the molecular mechanisms underlying the recruitment of pleural macrophages from the thoracic cavity to the lungs require further investigation.

Pleural macrophages, defined by immunostaining of Gata6, expressed Siglec-F in the inflamed lung tissue (**Figure 3B**). Siglec-F is a glycan-binding protein that is generally used as a surface marker to identify eosinophils, and pleural macrophages are known to be Siglec-F-negative in the thoracic cavity. However, there is emerging evidence that Siglec-F is expressed in cells other than eosinophils. For example, neutrophils expressing Siglec-F have recently been detected in various diseases. Siglec-F^high^ neutrophils accumulate in mouse lung adenocarcinoma and accelerate tumor growth through multiple mechanisms (54, 55). In a murine model of asthma induced by an air pollutant, DAMPs released from damaged tissue induced the expression of Siglec-F in neutrophils, and these cells exacerbated airway inflammation (56). It has been also demonstrated that Siglec-F is induced in bone marrow-derived macrophages upon stimulation with granulocyte-macrophage colony-stimulating factor (GM-CSF) to fine-tune macrophage characteristics (57). Furthermore, alveolar macrophages are also known to express high levels of Siglec-F in the lung. Thus, Siglec-F expression may be induced in pleural macrophages after their recruitment to inflamed lungs.

In conclusion, this study suggests that eosinophils and pleural macrophages are the cell types responsible for expressing 12/15-LOX in controlling eosinophilic airway inflammation in mice. These new findings indicate that 12/15-LOX-expressing eosinophils and pleural macrophages cooperatively regulate eosinophilic airway inflammation. Further studies are warranted to identify the regulatory mechanisms of 12/15-LOX metabolism in these cells and their modes of action in the regulation of airway inflammation. Therapeutic strategies to control 12/15-LOX expression and/or upregulate 12/15-LOX metabolism in these cells are expected to provide new methods for treating refractory eosinophilic diseases, especially severe asthma.

## Data Availability

The original contributions presented in the study are included in the article/**Supplementary Material**. Further inquiries can be directed to the corresponding authors.
