# Supplementary material for: Eosinophils and pleural macrophages counter regulate IL-33-elicited airway inflammation via the 12/15-lipoxygenase pathway
Source: Front Immunol. 2025 Apr 17;16:1565670. doi: 10.3389/fimmu.2025.1565670 (PMC12043891; doi:10.3389/fimmu.2025.1565670)
Supplement: Supplementary file 1 [file DataSheet1.pdf]

Supplementary Figure 1

No airway inflammation was observed in Alox15$^{fl/fl}$ and systemic 12/15-LOX deficient mice following PBS administration.

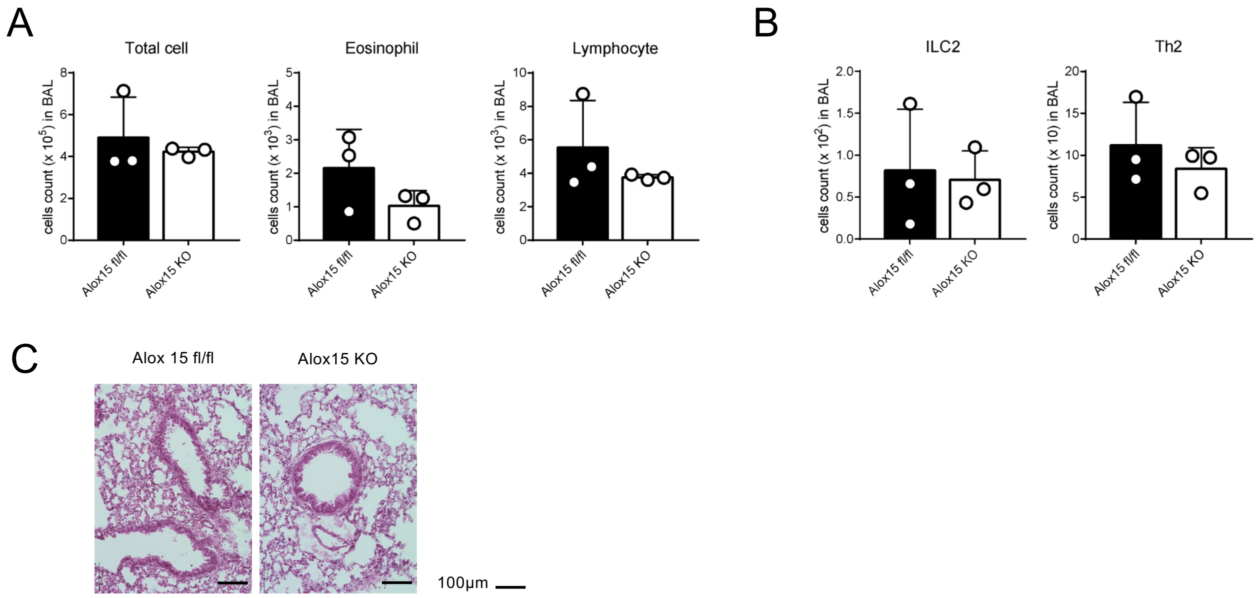

Supplementary Figure 1. No airway inflammation was observed in Alox15$^{fl/fl}$ and systemic 12/15-LOX deficient mice following PBS administration. Alox15$^{fl/fl}$ and systemic 12/15-LOX-deficient mice (Alox15 KO) were administered PBS intranasally for three consecutive days. Analysis was carried out four days after the final administration of PBS. (A) Number of total cells, eosinophils, and lymphocytes in BAL. (B) Number of ILC2 and Th2 cells in BAL was determined by flow cytometry. (C) Hematoxylin and Eosin staining of lung tissue sections.

Supplementary Figure 2

Genetic deletion of 12/15-LOX in mast cells, dendritic cells, and epithelial cells did not influence IL-33-induced airway eosinophilic inflammation.

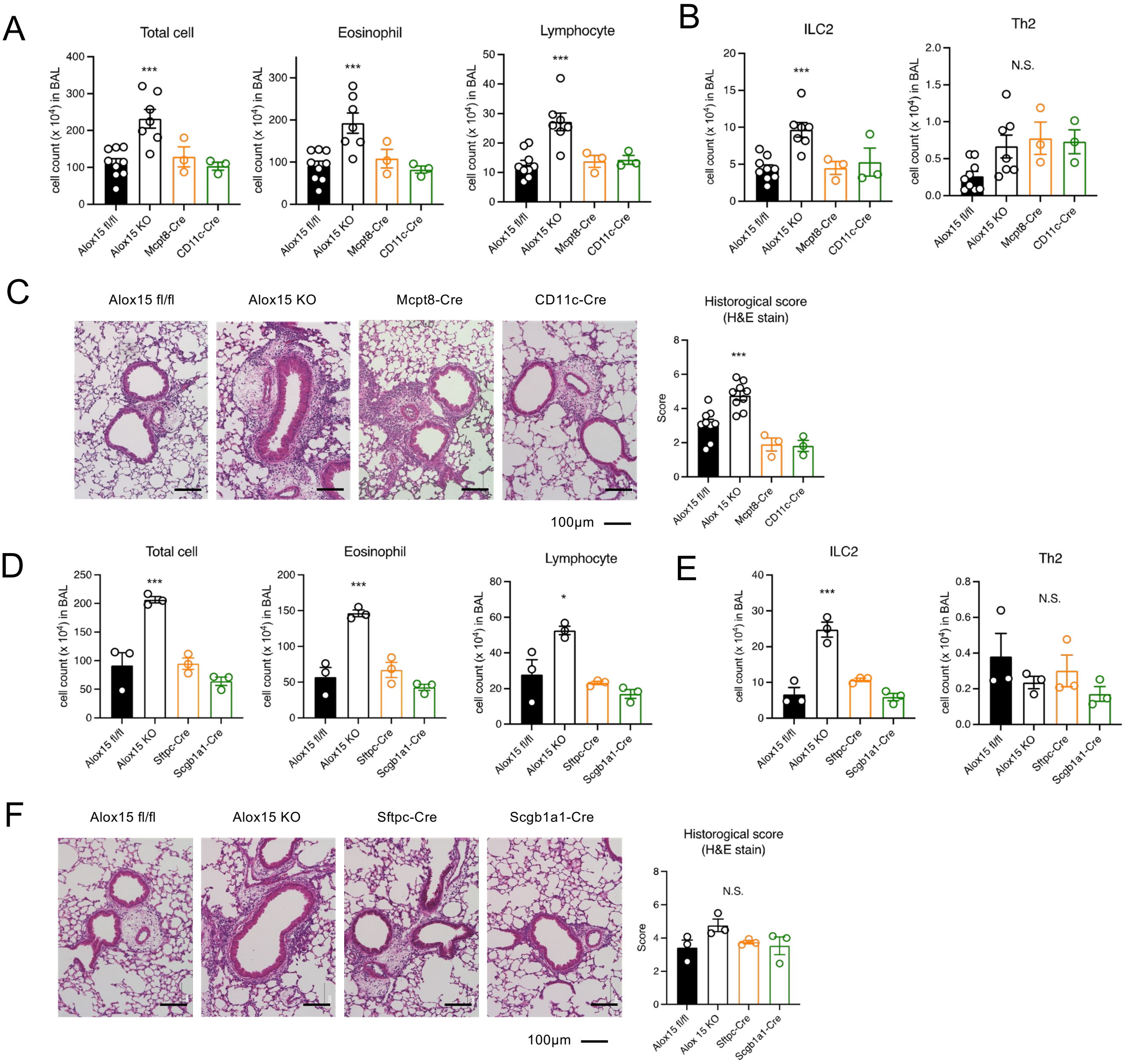

Supplementary Figure 2. Genetic deletion of 12/15-LOX in mast cells, dendritic cells, and epithelial cells did not influence IL-33-induced airway eosinophilic inflammation.

Airway inflammation was induced by intranasal administration of IL-33 (500 ng per mouse) for three consecutive days in C57BL/6 and 12/15-LOX-deficient mice (Alox15 KO) and cell-selective 12/15-LOX-deficient mice (Mcpt8-Cre Alox15$^{fl/fl}$: Mcpt8-Cre, CD11c-Cre Alox15$^{fl/fl}$: Cd11c-Cre, Sftpc-Cre Alox15$^{fl/fl}$: Sftpc-Cre, and Scgb1a1-Cre Alox15$^{fl/fl}$: Scgb1a1-Cre). Analysis was carried out four days after the final administration of IL-33. (A and D) Number of total cells, eosinophils, and lymphocytes in BAL. (B and E) Number of ILC2 and Th2 cells in BAL was determined by flow cytometry. (C and F) Hematoxylin and Eosin staining of lung tissue sections. Mean ± SEM, n=3-9 for each group. *P<0.05, **P<0.01, and ***P<0.001 compared with Alox15$^{fl/fl}$ mice.

Supplementary Figure 3

Pleural macrophages express 12/15-LOX under steady-state conditions.

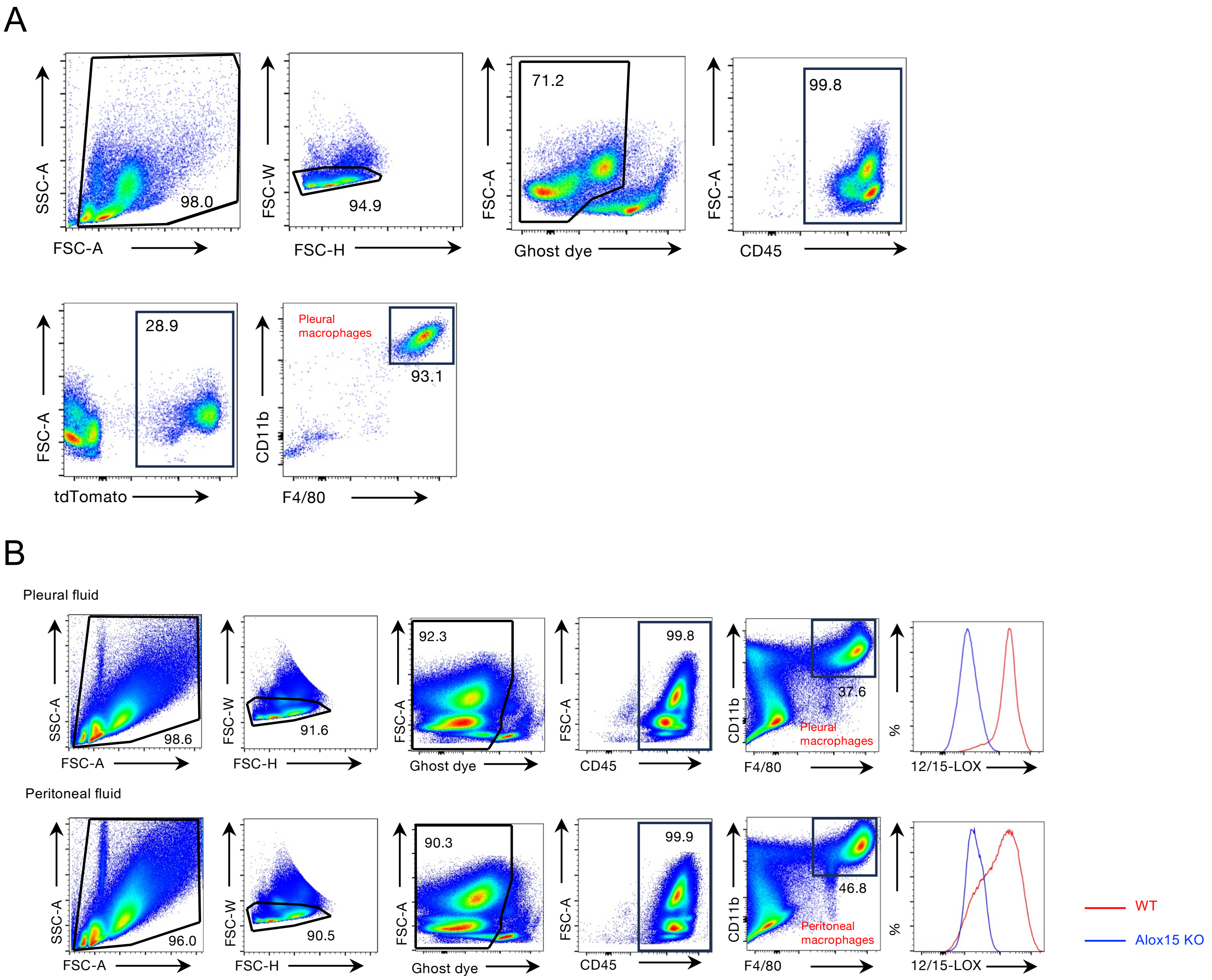

Supplementary Figure 3. Pleural macrophages express 12/15-LOX under steady-state conditions.

(A) Flow cytometric analysis of pleural cells in Alox15-Cre; Ai14 mice. (B) Flow cytometric analysis of the 12/15-LOX expression of cavity macrophages in pleural lavage fluid or peritoneal lavage fluid in wild-type mice.

Supplementary Figure 4 Pleural macrophages showed higher 12/15-LOX expression compared with eosinophils.

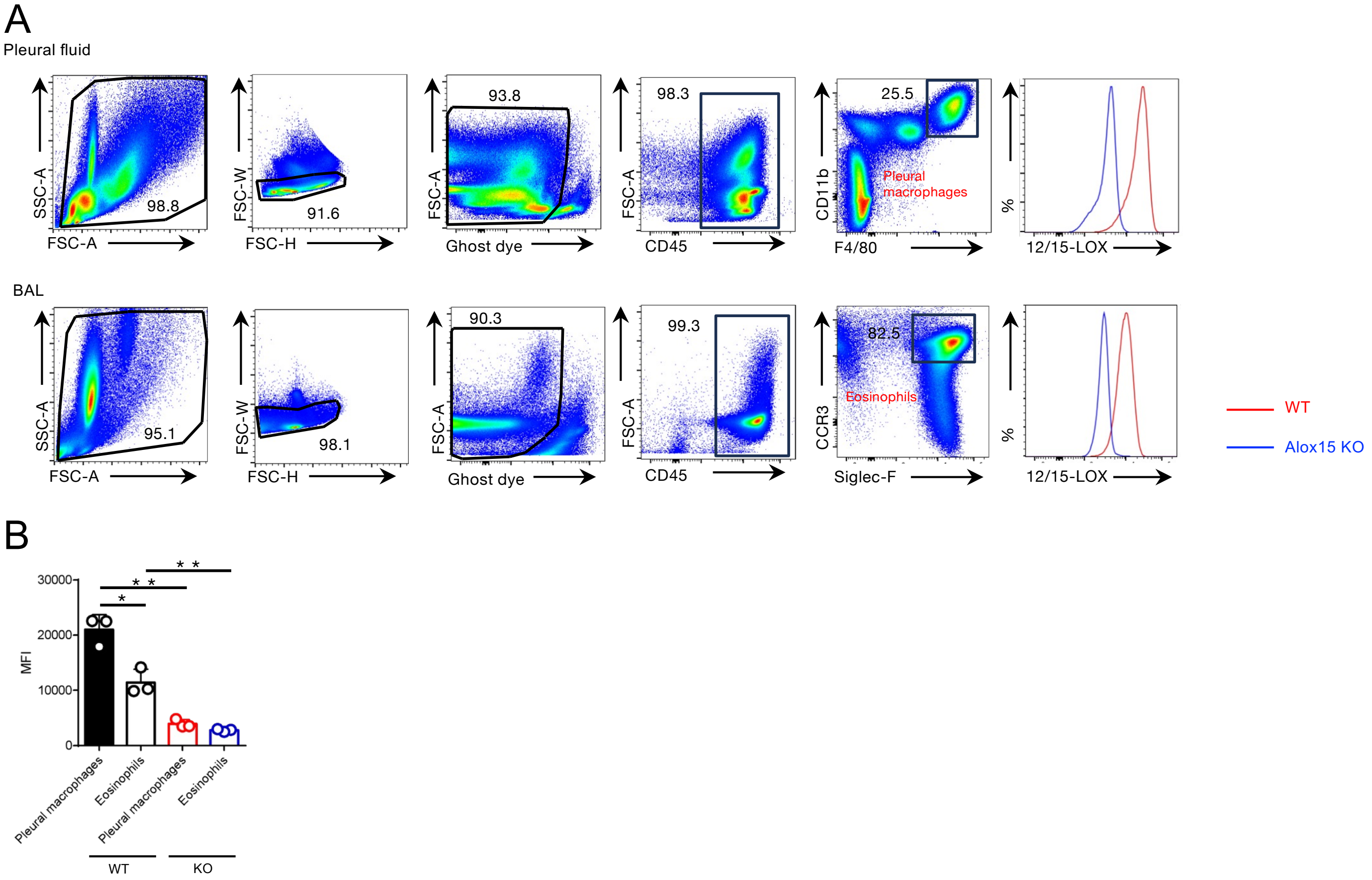

Supplementary Figure 4. Pleural macrophages showed higher 12/15-LOX expression compared with eosinophils.
(A) Flow cytometric analysis of the 12/15-LOX expression of pleural macrophages in PLF and eosinophil in BAL in wild-type and Alox15 KO mice. Analysis was carried out four days after the final administration of IL-33. (B) The mean fluorescent intensity (MFI) obtained by flow cytometric analysis. Mean ± SEM, n = 3. Data compared by one way ANOVA with Tukey's multiple comparison test (*P<0.05, **P<0.01).

Supplementary Figure 5 Pleural macrophages express Siglec-F and Gata6 in inflamed lung tissue.

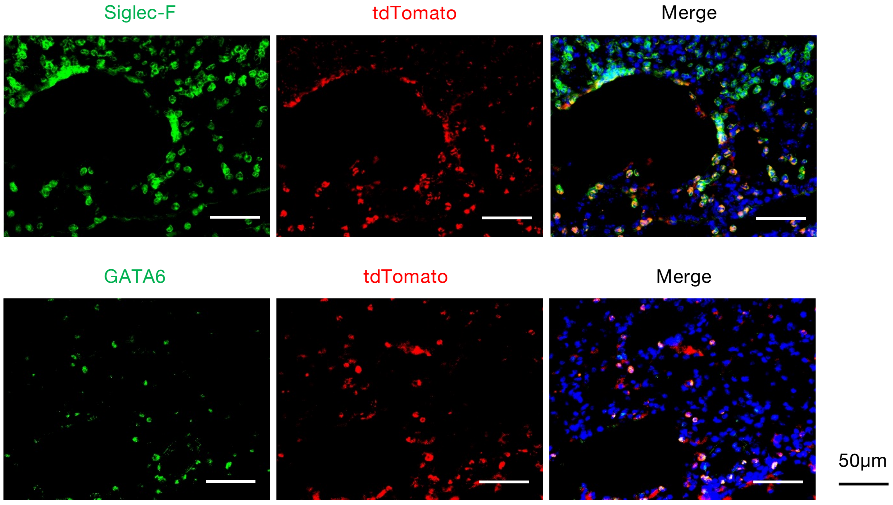

Supplementary Figure 5. Pleural macrophages express Siglec-F and Gata6 in inflamed lung tissue.

Lung tissue section of a mouse in which $tdTomato^{+}$ pleural macrophages were transferred, followed by IL-33 administration, were co-stained with Siglec-F and Gata6.
